# The effects of the adenosine A3 receptor agonist IB-MECA on sodium taurocholate-induced experimental acute pancreatitis

**DOI:** 10.1007/s12272-013-0126-2

**Published:** 2013-04-27

**Authors:** Beata Prozorow-Krol, Agnieszka Korolczuk, Grazyna Czechowska, Maria Slomka, Agnieszka Madro, Krzysztof Celinski

**Affiliations:** 1 Department of Gastroenterology with Endoscopic Unit, Medical University of Lublin, Jaczewski Street 8, 20-954 Lublin, Poland; 2 Department of Clinical Pathomorphology, Medical University of Lublin, Lublin, Poland

**Keywords:** Adenosine A3 receptors, Taurocholate acute pancreatitis, A3 agonist, Histology, α-Amylase, Lipase

## Abstract

The role of adenosine A3 receptors and their distribution in the gastrointestinal tract have been widely investigated. Most of the reports discuss their role in intestinal inflammations. However, the role of adenosine A3 receptor agonist in pancreatitis has not been well established. The aim of this study is (Ed note: Purpose statements should be in present tense) to evaluate the effects of the adenosine A3 receptor agonist on the course of sodium taurocholate-induced experimental acute pancreatitis (EAP). The experiments were performed on 80 male Wistar rats, 58 of which survived, subdivided into 3 groups: C—control rats, I—EAP group, and II—EAP group treated with the adenosine A3 receptor agonist IB-MECA (1-deoxy-1-6[[(3-iodophenyl) methyl]amino]-9H-purin-9-yl)-*N*-methyl-B-d-ribofuronamide at a dose of 0.75 mg/kg b.w. i.p. at 48, 24, 12 and 1 h before and 1 h after the injection of 5 % sodium taurocholate solution into the biliary–pancreatic duct. Serum for α-amylase and lipase determinations and tissue samples for morphological examinations were collected at 2, 6, and 24 h of the experiment. In the IB-MECA group, α-amylase activity was decreased with statistically high significance compared to group I. The activity of lipase was not significantly different among the experimental groups but higher than in the control group. The administration of IB-MECA attenuated the histological parameters of inflammation as compared to untreated animals. The use of A3 receptor agonist IB-MECA attenuates EAP. Our findings suggest that stimulation of adenosine A3 receptors plays a positive role in the sodium taurocholate-induced EAP in rats.

## Introduction

Acute pancreatitis (AP) remains a serious clinical problem. The pathogenesis of experimental acute pancreatitis (EAP) has been investigated for years. In 75–80 % of patients, the process takes the form of interstitial inflammation and is self-limited. In the remaining 20 %, the severe necrotizing-hemorrhagic course is observed with high overall mortality (Gravante et al. 2009). The severe form of EAP produces four types of morphological changes, i.e. proteolytic damage to the pancreatic interstitium, necrosis of vascular walls and hemorrhagic exudates, necrosis of the fatty tissue and inflammatory response. In the early stages, interstitial tissue edema develops which is a reversible condition that can lead to organ fibrosis and hyalinization. With progression, the areas affected by necrosis appear in both the endo- and exocrine parts of the pancreas and are accompanied by neutrophil infiltrates and interstitial hemorrhages. Necrosis of intra- and peripancreatic fatty tissue, called Balser’s necrosis, is most typical. With H + E staining, the necrotized adipocytes appear as pink homogenous cellular shadows, sometimes with basophilic deposits of calcium. In the course of EAP, necrotic foci of the adipose tissue can appear in the immediate vicinity of the pancreas and in other areas of the body, e.g. the omentum, mesentery of the colon, abdominal wall, or even the subcutaneous tissue (Gravante et al. 2009).

Adenosine is an endogenous nucleoside present in all the body cells that is involved in important physiological and pathological processes. 5′-AMP and S-adenosylhomocysteine are the basic sources of adenosine (Romanowska and Komoszynski 2002). The major activity of adenosine is accomplished by specific P1 membranous receptors. All subtypes of adenosine receptors are found in the gastrointestinal tract, e.g. A1, A2a, A2b, and A3. A1 and A2 receptors exhibit high affinity to adenosine while A2b and A3 receptors have substantially lesser affinity (Romanowska and Komoszynski 2002; Dunwiddie et al. 1997). A1, A2, and A3 receptors are unevenly distributed in the tissues. More than one adenosine receptor subtype can be expressed in the same cell (Romanowska and Komoszynski 2002; Dunwiddie et al. 1997). Individual receptor subtypes play different roles. Therefore, elucidation of mechanisms of signal transmission with adenosine and P1 involvement may be valuable in clinical practice.

The A3 receptor subtype has been the latest P1 receptor identified. It is known that its stimulation leads to inositol triphosphate formation and results in increased calcium concentration in the cytoplasm (Dunwiddie et al. 1997).

The role of adenosine A3 receptors and their distribution in the gastrointestinal tract have been widely investigated. Most of the reports discuss their role in intestinal inflammations. In the pancreas, A3 might be located in the vascular endothelial cells and within nerve fibers (Celinski et al. 2006). A3 receptors are supposed to regulate the A1 receptor activity (Dunwiddie et al. 1997). Among all of the adenosine receptors, the role of A3 and its agonist IB-MECA is least elucidated, particularly in acute necrotizing pancreatitis. Sodium taurocholate-induced acute pancreatitis is the best experimental model reflecting the course of this inflammation.

The purpose of this experimental study is to determine the effects of IB-MECA, the adenosine A3 receptor agonist, on histopathological changes and amylase as well as lipase activity in sodium taurocholate-induced experimental acute pancreatitis. Due to its pioneering nature, the present study is limited to the determinations of activities of pancreatic enzymes and histopathological examinations, yet our intension is to widen the scope of further studies with pro-inflammatory cytokines or mRNA expression.

## Materials and methods

The present study is a continuation of the research carried out by our team in 2002–2003. The examinations were conducted on 80 white male Wistar rats weighing 250–300 g; 58 of rats survived.

The animals were divided into three experimental groups.

Group C—the control healthy animals used to determine the biochemical norms and standard histological appearance. The rats were administered 1 ml 0.9 % NaCl i.p. (*n* = 10).

Group I—the animals in which EAP was induced by injecting 5 % sodium taurocholate solution (Sigma Chemical Co.) at a dose of 0.08 ml/100 g b.w. into the biliary–pancreatic duct according to Aho and Henckel (Aho et al. 1983) and 1 ml 0.9 % NaCl i.p. (*n* = 38/survived 23). The mortality rate = 39.5 %.

Group II—the rats with EAP administered IB-MECA A3 receptor agonist (Tocris Cookson Ltd.) at a dose of 0.75 mg/kg b.w. i.p. (Mabley et al. 2003) at 48, 24, 12 and 1 h before and 1 h after the injection of 5 % sodium taurocholate solution into the biliary–pancreatic duct (*n* = 32/survived 25). The mortality rate = 21.87 %.

After 2, 6 and 24 h the animals were anaesthetized with diazepam (0.15 mg/kg b.w.) and ketamine (5 mg/kg b.w.). The blood samples were collected from the left ventricle for biochemical tests. Samples of the pancreas were obtained for histopathological examinations. The study design was approved by the Local Bioethical Committee of the Medical University of Lublin.

### Biochemical assays

The blood samples were collected from the left ventricle, centrifuged at 3,000×*g* for 10 min at 4 °C and collected for further analysis. The activities of α-amylase and lipase in the blood serum were determined using the standard laboratory methods as well as AMYL and LIPA Vitros 250 systems (USA).

### Histological examination

For histological examinations, the pancreatic sections were fixed in 10 % buffered formalin solution, pH 7.4. The sections were embedded in paraffin and cut into 2 pm-thick slices using a microtome. The specimens were stained with hematoxylin and eosin (H&E).

Histological features of pancreatitis, i.e. necrotic lesions in the parenchyma and adipose tissue of the pancreas, inflammatory infiltrations, erythrocyte extravasations and hemorrhages, and interstitial tissue edema were assessed. The scale according to Satoh et al. (2002) was used to evaluate morphological lesions in the individual groups. Histological changes of the pancreas were graded blindly (range 0–4) based on the approximate percentage of the acinar cells showing vacuolization and necrosis, interstitial edema and the approximate areas showing inflammatory cell infiltration and hemorrhage: 0 absent, 1 <5 %, 2 5–25 %, 3 25–50 %, 4 >50 % (Celinski et al. 2006; Satoh et al. 2002).

### Statistical analysis

The values of biochemical parameters were statistically analyzed. The results were expressed as the mean ± standard error of the mean (mean ± SEM). The data were compared using the analysis of variance (ANOVA). A 5 % risk of inference error was accepted; *p* < 0.05 was considered statistically significant. Histological data were expressed as the range of score and the mean ± SE and compared using the Student’s test (*p* < 0.05 was considered statistically significant).

## Results

Statistically higher significant activities of α-amylase were observed in the group treated with sodium taurocholate in comparison to the control group (*p* < 0.05). These activities decreased after the administration of IB-MECA (Table 1) (*p* < 0.01). Activities of lipase were slightly higher after sodium taurocholate compared to the control group (ns) and were not significantly different from the group pretreated with IB-MECA (Table 2) (ns).

**Table 1:** The effects of adenosine A3 agonist on the serum α-amylase activity in experimental acute taurocholate pancreatitis in rats (EAP)

Group	Time (h)	Mean	SEM	Me	ΔMe	ANOVA	*p*
Control (C)		569	61.7	584			
Sodium taurocholate (EAP)	2	2,835	588	2,679	2,095	16.2	*p*_1_ < 0.05
	6	2,069	558	1,962	−717		
	24	2,603	248	2,530	568		
EAP + Agonist A3 (IB-MECA)	2	1,627	165	1,640	1,056	13.0	*p*_2_ < 0.01
	6	1,487	212	1,533	−106		
	24	2,218	293	2,068	535		

*p*-Statistical significance of differences in comparison to: control group *p*_1_ < 0.05, EAP *p*_2_ < 0.01

*SEM* standard error of the mean, *Me* median, *ΔMe* delta median, *ANOVA* analysis of variance

**Table 2:** The effects of adenosine A3 agonist on the serum lipase activity in experimental acute taurocholate pancreatitis in rats (EAP)

Group	Time (h)	Mean	SEM	Me	ΔMe	ANOVA	*p*
Control (C)		24.0	7.48	22.0			
Sodium taurocholate (EAP)	2	34.4	8.20	36.0	14.0	5.0	ns
	6	34.8	11.8	31.5	−4.50		
	24	41.3	17.5	34.5	3.0		
EAP + Agonist A3 (IB-MECA)	2	35.8	16.3	28.0	6.0	1.75	ns
	6	49.1	35.8	42.0	14.0		
	24	32.7	15.2	23.5	18.5		

*SEM* standard error of the mean, *Me* median, *ΔMe* delta median, *ANOVA* analysis of variance, *ns* not significant

### Histological changes

**Fig. 1:**
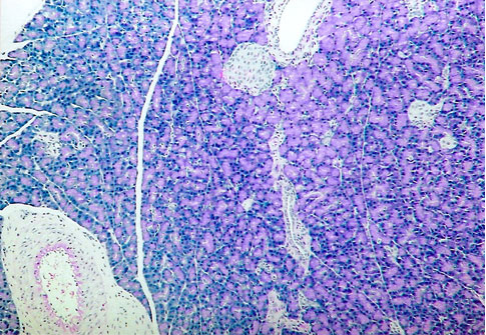
Group C. H + E ×100. Normal pancreatic lobule

Group C: Normal histological pancreatic structure was found after the i.p. administration of 1 ml 0.9 % NaCl (Fig. 1).

**Fig. 2:**
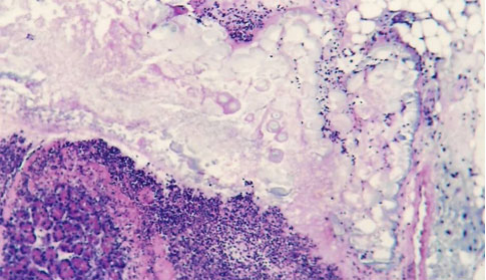
Group I. H + E ×200. Enzymatic necrosis of peripancreatic adipose tissue and necrosis of the pancreatic parenchyma with dense inflammatory infiltrate

**Fig. 3:**
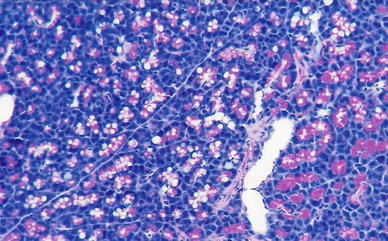
Group I. H + E ×200. Vacuolar degeneration of parenchymal cells of the pancreas in the areas not involved by necrosis

In group I, animals developed typical microscopic appearance of EAP over 24 h of the experiment. Microscopic examination showed extensive necrosis of the exocrine parenchyma, intensive interstitial edema and focal inflammatory infiltrates of various intensities consisting of neutrophils and scattered lymphocytes. This process spread to the peripancreatic adipose tissue in the form of enzymatic necrosis (Fig. 2). Damaged walls of the intrapancreatic ducts with necrosis of the epithelium of the pancreatic parenchyma were observed. Moreover, vacuolar degeneration of the cytoplasm was found in the necrotic cells (Fig. 3; Table 3).

**Table 3:** Histopathological findings of the pancreas in rat treated with EAP in comparison to control group

Time (h)	*N*	Inflammation	Necrosis	Hemorrhage	Vacuolization	Edema
Control						
	10	0–1	0	0	0	0–1
		0.22 ± 0.03	0.00 ± 0.00	0.00 ± 0.00	0.00 ± 0.00	0.14 ± 0.04
EAP						
2	8	1	1	1–2	1	1
		0.23 ± 0.04*	0.01 ± 0.01^**^	1.21 ± 0.05^***^	0.11 ± 0.03^**^	0.15 ± 0.03*
6	8	2	2–3	2–3	2–3	2–3
		1.77 ± 0.06^***^	2.34 ± 0.05^***^	2.68 ± 0.09^***^	2.74 ± 0.05^***^	2.59 ± 0.09^***^
24	7	–4	4	4	3–4	3–4
		3.52 ± 0.06^***^	3.89 ± 0.07^***^	3.81 ± 0.08^***^	3.32 ± 0.08^***^	3.62 ± 0.08^***^
EAP + IB-MECA						
2	9	0–1	0–1	1	0	0
		0.11 ± 0.02	0.43 ± 0.05^***^	0.56 ± 0.03^***^	0.00 ± 0.00	0.00 ± 0.00^***^
6	8	0–1	2	1	0–1	0
		0.58 ± 0.02^***^	1.47 ± 0.06^***^	0.94 ± 0.04^***^	0.14 ± 0.05^***^	0.00 ± 0.00^***^
24	8	1–2	2	1–2	1–2	0–1
		1.78 ± 0.07^***^	1.92 ± 0.05^***^	1.39 ± 0.06^***^	1.48 ± 0.04^***^	0.67 ± 0.01^***^

Grading of microscopic changes was based on the approximate percentage of the pancreatic tissue involved: inflammation, necrosis, hemorrhage, vacuolization and edema as follows: 0: absent, 1: <5 %, 2: 5–25 %, 3: 25 > 50 %, 4: >50 %

* *p* > 0.05, ** *p* < 0.01, *** *p* < 0.001

**Fig. 4:**
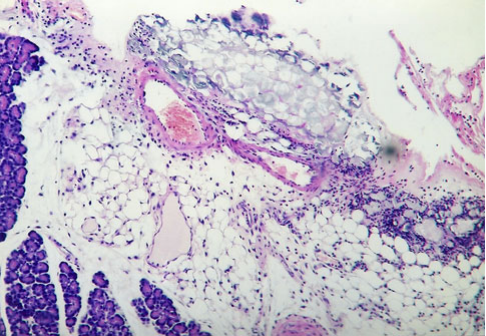
Group II. 2 h after induction of pancreatitis. H ± E ×200. Focal enzymatic necrosis of the adipose tissue with the presence of typical calcium soaps

**Fig. 5:**
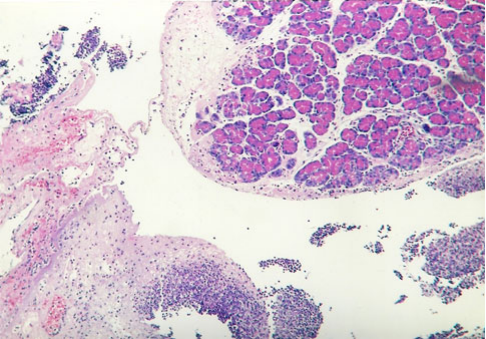
Group II. 6 h after induction of pancreatitis. H + E ×100. Inflammatory lesions confined to the lobular periphery. Focal features of parenchymatous degeneration of acinar cells. Slight inflammatory infiltrate composed of neutrophils. Necrotic cells deprived of nuclei on the periphery. Inflammatory–necrotic lesions in the adipose tissue

**Fig. 6:**
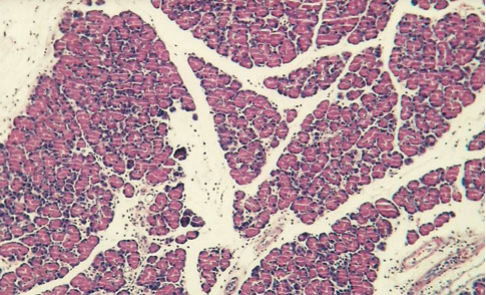
Group II. 24 h after induction of pancreatitis. H + E ×200. Interstitial edema of the pancreas accompanied by mild inflammatory infiltrate composed mostly of lymphocytes

In group II treated with IB-MECA 2 and 6 h after 5 % sodium taurocholate injection, animals developed interstitial edema, slightly intense inflammatory infiltrates, erythrocyte extravasations into the adipose and interstitial tissue and foci of blurred parenchymal lobular cells (Figs. 4, 5); at 24 h after 5 % sodium taurocholate administration, deliquescent necrosis was developed which was accompanied by erythrocyte extravasations and inflammatory cells. Moderate intralobular edema and focal vacuolization of parenchymal cells were observed (Fig. 6; Table 3).

The effects of adenosine A3 receptor agonist on the microscopic findings of EAP and EAP + IB-MECA are presented in Table 3.

## Discussion

The role of adenosine in the gastrointestinal tract has been investigated since the 1960s and the research has been focused on the effects of adenosine on the blood flow, motor activity and gastric secretion. There are, however, reports on adenosine involvement in certain pathological conditions (Dunviddie et al. 1997; Noji et al. 2002; Lee et al. 2006; Novak et al. 2008).

The purpose of the present experimental study is to investigate the effects of the adenosine receptor A3 agonist on the course of EAP. As mentioned earlier, A3 subtype receptors have been most recently discovered and thus, limited knowledge of this P1 receptors. In the pancreas, A3 receptors are likely to be found in the vascular endothelial cells and within nerve fibers (Satoh et al. 2002).

Novak et al. studied adenosine receptor distribution in the pancreatic ducts in rats. They found that the concentration of receptor subtypes was A2A > A2B > A3 ≫ A1 (Novak et al. 2008). A3 receptors show substantially low affinity to adenosine; to be activated, they require agonist’s concentration over 1^M. Such a high concentration of extracellular ecto-adenosine, exceeding the upper physiological limit, is often determined in tissue anoxia (Novak et al. 2008).

In our experiment, IB-MECA—the A3 receptor agonist (group II)—reduced morphological changes compared to the untreated animals (group I). We observed 30 % damage to the pancreatic parenchyma. After 24 h from the onset of inflammation, the preparations demonstrated moderate edema. Thus, it can be stated that in the group of animals pretreated with IB-MECA the intensity of inflammation and edema decreased, compared to group I. In both groups biochemical scores were similar (Tables 1, 2).

A slight reduction of inflammation due to IB-MECA present in our experiment was also reported by Mabley et al. in the experimental intestinal inflammation in rats (Mabley et al. 2003). In their study, IB-MECA decreased the number of inflammatory cellular infiltrates during intestinal inflammation and normalized the level of cytokines and chemokines, especially interleukin-10. IL-10 is said to be specifically involved in modulating the course of EAP. According to Osman and Jensen (1999), IL-10 agonist administered as the prophylactic protocol in necrotizing EAP reduced the mortality rates and decreased the development of acute damage to the lung tissue in rats. Lee et al. (2006) suggested that IB-MECA administered in experimental bacterial peritonitis in guinea pigs reduced the mortality and hepatorenal complications due to decreased production of proinflammatory cytokines, chemokines and TNF-α. The observation is confirmed by our results noted 24 h after the onset of inflammation.

Moreover, Smith et al. (2002) investigated the influence of A3 receptor agonist IB-MECA and A2 receptor agonist CGS 21680 on experimental endotoxemia in rats. They found that both agents demonstrated anti-inflammatory activities and reduced the production of IL-10 and TNF-α.

TNF-α is responsible for multi-organ damages in EAP. In the pancreas, TNF-a is mainly produced by inflammatory leukocytes located in the infiltrates (Mc Whinney et al. 1997). According to Fishman and Bar-Yehuda (2003), anti-inflammatory activity of adenosine can be accomplished via A3 receptors found in neutrophils, eosinophils and macrophages by controlling the degranulation or cytokine production. Moreover, basophils display high expression of A3 receptors yet their degranulation leads to pro-inflammatory activity.

Abbrachio et al. (1995) postulated that A3 receptors were likely to induce cellular apoptosis under stress conditions. In sodium taurocholate-induced necrotizing-hemorrhagic EAP, increased necrosis and decreased apoptosis were observed. Dlugosz et al. investigated the effects of stable prostacyclin analogue iloprost on the trypsinogen activation, labilization of lysosomal membranes, activities of lipolytic enzymes, histopathological and ultrastructural changes in the pancreas of rats with severe, taurocholate acute pancreatitis (AP), preceded by acute ethanol intake for 6 h. Treatment with iloprost limited the labilization of lysosomal membranes in non-alcoholized rats with AP. The protective effect of iloprost in AP is thought to be dependent on the attenuation of trypsinogen activation, decrease in total trypsin potential and reduction in lysosomal membrane labilization. Its protective effect could be limited in taurocholate acute pancreatitis preceded by acute ethanol intake as evidenced by the differences in the cathepsin B, phospholipase A2 and lipase activities and by histopathological and ultrastructural findings (Dlugosz et al. 2004). Molecular mechanism involved in EAP attenuation needs further investigations.

Short-term stimulation of A3 receptors decreased the blood flow through the tissues and increased damage; otherwise, long-term stimulation led to increased circulation and decreased pathological changes (Romanowska and Komoszynski 2002). Numerous studies suggest that A3 receptor agonists in vivo decrease the damage caused by anoxia. Zhao et al. (1993) have found that A3 receptor activation protects the heart from damage during ischemia. Stimulation of the adenosine A3 receptor modulates mitogen-activated protein kinase (MAPK) activity through various mechanisms (Shulte and Fredholm 2003) and induces ERK (extracellular-signal-regulated kinase) activation in newborn rat cardiomyocytes, which imply a potential role of MAPK in adenosine A3 receptor induced cardioprotection (Germack and Dickenson 2004) Dunwiddie et al. (1997) have demonstrated that A3 receptor activation in the hippocampus in rats desensitizes A1 receptors whose activity inhibits the secretion of stimulating neurotransmitters. IB-MECA, a selective A3 receptor agonist, exerts a significant influence on neuronal survival and cerebral blood flow in rats. Since A3 receptors are also located in astrocytes (Brambilla et al. 2003) and in microglia cells (Hammarberg et al. 2003), it is likely that the impact of these receptors on neuronal damage might indirectly result from the control of the reactivity of the glia cells. In recent clinical study, van Troostenburg et al. assessed the safety, tolerability and pharmacokinetics of IB-MECA in healthy men. They found that a single oral dose of up to 5 mg of IB-MECA and repeated doses of up to 4 mg every 12 h for 7 days were safe and well tolerated. However, further dose increases were associated with flushing, tachycardia, nausea and vomiting (van Troostenburg et al. 2004).

The administration of IB-MECA can limit edema and inflammatory infiltrates and modulate the changes developing in the course of EAP. Numerous studies have confirmed favorable effects of adenosine and its analogues on termination of supraventricular tachycardia, decrease in hypertension, treatment of leukemia, renal diseases, asthma, Parkinson’s disease, Alzheimer’s disease and pain management. There is little information on adenosine applied in the treatment of gastrointestinal diseases, including the pancreas. More and more reports are being published on newly discovered aspects of adenosine activity and pathogenesis of EAP.
